# The Popularization of Mechanical Therapeutics

**Published:** 1903-04

**Authors:** 


### THE POPULARIZATION OF MECHANICAL THERAPEUTICS.

This is the age of mechanical therapeutics. The growing distrust in drugs which has for years been creeping over the profession causes a reaching out for something more substantial upon which to rest, and as a result light, electricity and systems of gymnastics are engrossing the attention of the profession. Pharmacology is in a measure pushed aside by the onrush of new ideas in the treatment of disease. This notion is perhaps prevalent outside the profession more than within, and promises to produce a new species of charlatanry; the exploitation of systems of self taught exercise. The advertising pages of the magazines are embellished with pictures of primitively clad gentlemen with lumpy muscles in gladiatorial poses, accompanied by the invitation to buy one of their systems and become like as they are. Of course this is more or less an innocent form of quackery. It uses no drugs, not even of the "purely vegetable" kind and is calculated to do more good than harm by teaching some lazy man or woman how to work. The element of quackery is contained in claiming to do much and alleging curative virtues to exercises of this kind which do not and in the natnre things can not exist. Without doubt, exercise is a useful adjunct to other forms of rational therapy, but there are many diseases in which it is inappropriate if not actually harmful. Of what use to the ordinary business or professional man is spectacular muscle building? It may help him in a way if his share of domestic duties include splitting kindling and bringing up coal, or carrying the offspring on the Sunday afternoon stroll; but lacking these cooperative influences, the slight muscular hypertrophy disappears when the exercises or the formulated movement is discontinued. The real benefit to be derived from exercise consists in bringing the organs essential to life into a more normal, physiological condition and thus restoring or tending to restore the equilibrium which has been disturbed by functional disease or faulty living. Working up a showy biceps is no better from the larger view of the purposes of exercise, than urging the growth of a wart on the nose. If a really intelligent system of exercise were popularized, we would even be willing for the quacks to reap the reward for the possible good that might accrue. No form of indoor gymnastics can quite fulfil the ideal. It cannot replace the deficiencies from deprivation of outdoor life with its slowly but surely conferred benefits. A brisk morning walk or a spin on a wheel is a more pleasing sacrifice to Hygeia than tugging at a resisting rubber band o describing geometric figures in the air with tired arms and legs.

